# Magnetoresistance Versus Oxygen Deficiency in Epi-stabilized SrRu_1 − *x*_Fe _*x*_O_3 − *δ*_ Thin Films

**DOI:** 10.1186/s11671-017-1950-y

**Published:** 2017-03-06

**Authors:** Umasankar Dash, Susant Kumar Acharya, Bo Wha Lee, Chang Uk Jung

**Affiliations:** Department of Physics and Oxide Research Centre, Hankuk University of Foreign Studies, Yongin, 17035 South Korea

**Keywords:** Epitaxial thin film, X-ray diffraction, Reciprocal space mapping, Oxygen vacancy, Magnetoresistance, Fe-doped SrRuO_3_

## Abstract

Oxygen vacancies have a profound effect on the magnetic, electronic, and transport properties of transition metal oxide materials. Here, we studied the influence of oxygen vacancies on the magnetoresistance (MR) properties of SrRu_1 − *x*_Fe_*x*_O_3 - *δ*_ epitaxial thin films (*x* = 0.10, 0.20, and 0.30). For this purpose, we synthesized highly strained epitaxial SrRu_1 − *x*_Fe_*x*_O_3 − *δ*_ thin films with atomically flat surfaces containing different amounts of oxygen vacancies using pulsed laser deposition. Without an applied magnetic field, the films with *x* = 0.10 and 0.20 showed a metal–insulator transition, while the *x* = 0.30 thin film showed insulating behavior over the entire temperature range of 2–300 K. Both Fe doping and the concentration of oxygen vacancies had large effects on the negative MR contributions. For the low Fe doping case of *x* = 0.10, in which both films exhibited metallic behavior, MR was more prominent in the film with fewer oxygen vacancies or equivalently a more metallic film. For semiconducting films, higher MR was observed for more semiconducting films having more oxygen vacancies. A relatively large negative MR (~36.4%) was observed for the *x* = 0.30 thin film with a high concentration of oxygen vacancies (*δ* = 0.12). The obtained results were compared with MR studies for a polycrystal of (Sr_1 − *x*_La_*x*_)(Ru_1 − *x*_Fe_*x*_)O_3_. These results highlight the crucial role of oxygen stoichiometry in determining the magneto-transport properties in SrRu_1 − *x*_Fe_*x*_O_3 − *δ*_ thin films.

## Background

The electronic and magnetic properties of SrRuO_3_ (SRO) have received a significant amount of attention, not only from a fundamental science point of view but also due to their potential for device applications [1–3]. In particular, SRO in its thin film form is widely used in oxide electrodes due to its favorably high conductivity, atomically flat surface, and low lattice mismatch with single-crystalline perovskite oxide substrates [4, 5]. SRO is an itinerant ferromagnetic metal that shows a transition from the paramagnetic to ferromagnetic state at the Curie temperature (*T*_*C*_) = 163 K for polycrystalline samples [1, 6]. The ferromagnetism in SRO originates from a substantial spin polarization of the low-spin-state configuration of the 4*d* electrons (S = 1; *t* ^4^_2*g*_ *e* ^0^_*g*_), producing a saturated magnetic moment of 1.6 μ_*B*_ [7]. Bearing in mind that the itinerant ferromagnetism of SRO originates from a narrow *t*_2*g*_ band, the bandwidth can be dramatically modified by doping 3*d* metal ions such as Ti^4+^, Cr^3+^, Mg^2+^, Ni^2+^, and Fe^3+^, giving rise to exotic magnetic and transport properties [8, 9].

Certain fascinating doping effects have been reported to arise from the substitution of Fe^3+^ ions at the Ru^4+^ sites in SRO [10, 11]. For instance, Mamchik et al. observed a “self-spin valve” large negative magnetoresistance (MR) in polycrystalline (Sr_1 − *x*_La_*x*_)(Ru_1 − *x*_Fe_*x*_)O_3_ (SLRFO) samples [12]. In their study, the maximum MR (~45%) was reported for (Sr_0.7_La_0.3_)(Ru_0.7_Fe_0.3_)O_3_ samples at *T* = 10 K and *H* = 9 T. They proposed that the large negative MR was due to the ferromagnetic interaction between the high-spin state of Fe^3+^ ions and low-spin state of Ru^4+^ ions. Co-doping of La^3+^ at the A sites was critical for stabilization of the single phase in their polycrystalline samples. In contrast, we recently showed that high-quality Fe-doped SRO without A-site co-doping can be stabilized using an epitaxial strain during thin film growth [13, 14]. By preparing highly crystalline epitaxial thin films, we also avoided effects arising from the porosity and grain boundaries of polycrystalline samples. In that work, we fabricated SrRu_1 − *x*_Fe_*x*_O_3_ (SRFO) thin films with various Fe doping levels (*x* = 0.05, 0.10, and 0.20) and observed a maximum negative MR of 14.4% for a 10% Fe-doped SRFO epitaxial thin film at *T* = 10 K and *H* = 9 T [13].

However, the maximum MR value of ~14.4% in our previous epitaxial thin film was substantially less than that of co-doped SLRFO polycrystalline samples (45%). Obtaining a large MR is an essential goal for various technological applications, including data storage, non-volatile memory, and sensing applications [15, 16]. Thus, we searched various options to improve the MR values in our epitaxial SRFO thin films. In particular, we noticed that in polycrystalline SLRFO samples, the MR was higher for more semiconducting samples [12]. In contrast, our SRFO thin films with *x* = 0.00, 0.05, 0.10, and 0.20 with intentionally reduced levels of oxygen vacancies were mostly metallic [13]. Oxygen vacancies, which are intrinsic to perovskite oxides, play a major role in the electronic and magnetic properties of strongly correlated systems. These vacancies effectively act as double electron donors, tuning the valence states of B-site transition metal cations in ABO_3_ perovskite oxides [17]; as a consequence, the magneto-transport properties of these films are strongly impacted [18]. For instance, in La_0.5_Sr_0.5_CoO_3 − *x*_ perovskite thin films, the negative MR value sharply increased from 1 to 17% when the oxygen partial pressure during the thin film growth changed from 1 to 0.1 mbar [19]. (Even though the oxygen vacancy was not calculated quantitatively, the observed lattice expansion was suggested to be an indicator of an increase in oxygen vacancies.) Similarly, in La_0.7_Sr_0.3_MnO_3_ thin films, the MR increased from 55 to 85% when the thin films were deoxygenated by annealing in a reducing atmosphere [20]. Thus, a semiconducting SRFO thin film with increased oxygen-vacancy content is expected to show higher MR than a metallic SRFO thin film.

In this study, we estimate the oxygen-vacancy content in SrRu_1 − *x*_Fe_*x*_O_3 − *δ*_ (*x* = 0.10, 0.20, and 0.30) thin films deposited at various oxygen partial pressures and examine the impact of oxygen vacancies on MR and transport properties. In addition, we also report a high negative MR of ~36.4% observed in semiconducting SrRu_0.7_Fe_0.3_O_3 − 0.12_ epitaxial thin films. This MR value for thin films of SrRu_0.7_Fe_0.3_O_3 − 0.12_ with a high level of oxygen vacancies is more than two times that of the previously reported highest MR (~14.4%) value for low-oxygen-vacancy thin films of SrRu_0.9_Fe_0.1_O_3 −0.02_ [13].

## Methods

The current study involved three sets of samples: (1) We reviewed the reported MR and structural analysis for previously reported low-oxygen-vacancy thin films [13], (2) we measured the MR of the high-oxygen-vacancy thin films used in our previous report [14], and (3) we fabricated new SRFO thin films with high concentrations of oxygen vacancies. These SrRu_1 − *x*_Fe_*x*_O_3 − *δ*_ (*x* = 0.10, 0.20, and 0.30) thin films with different oxygen vacancies were grown on SrTiO_3_ (STO) substrates under different oxygen partial pressures using pulsed laser deposition (PLD). The details of film growth can be found in our previous reports [13, 14, 21, 22]. The laser power and substrate temperature were maintained at 35 mJ and ~750 °C, respectively, at a constant frequency of 4 Hz. During thin film growth, the oxygen partial pressure was set at either 100 or 180 mTorr. Therefore, it is expected that the oxygen content differs among the thin films. The thickness of the films was characterized by a surface profilometer. The quality of the thin films was studied in multiple ways, including the lattice parameter, lattice volume, and surface morphology. The crystal structure was characterized by high-resolution X-ray diffraction (HRXRD). (Two thin films were characterized using the Bruker D8 Discover HRXRD system, while the other three films were characterized with the PANalytical X’Pert PRO HRXRD system.) The surface morphology was examined by atomic force microscopy (AFM). MR was measured by using a cryogen-free cryostat (CMag Vari9, Cryomagnetics Inc.) and a dual-channel source-measure unit (Keithley 2612A Standard Measurement Unit) [13].

## Results and Discussion

In our previous two reports, we showed that phase-pure high-quality epitaxial SrRu_1 − *x*_Fe_*x*_O_3 − *δ*_ (*x* = 0.1, 0.2, 0.3) thin films with either high or low oxygen-vacancy content can be grown on STO (001) substrates [13, 14]. In the current study, we selected 10 and 20% Fe-doped SRFO thin films deposited at pressures of 100 and 180 mTorr and grew new 30% Fe-doped SRFO thin films at a pressure of 100 mTorr.

**Fig. 1:**
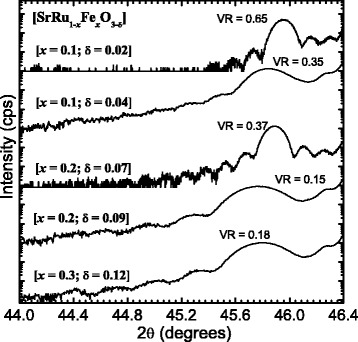
X-ray diffraction patterns of SrRu_1-*x*_Fe_*x*_O_3-*δ*_ (0.10  ≤ *x* ≤ 0.30) epitaxial thin films. (Note that the 2*θ* patterns of two films were taken with the Bruker D8 Discover system [13] while those of the other three were taken with the PANalytical X’Pert PRO HRXRD system [14], which resulted in different background signals.)

Figure 1 presents the HRXRD patterns of these series of SrRu_1 − *x*_Fe_*x*_O_3 − *δ*_ (*x* = 0.1, 0.2, and 0.3) epitaxial thin films. The figure shows only the local range centered at the (002) reflections of the SRFO thin films. For simplicity, all reflection peaks are indexed in the tetragonal notation for the crystal structure of the SrRu_1 − *x*_Fe_*x*_O_3 − *δ*_ films, and the HRXRD intensity is plotted using a log scale [13, 14]. The rocking curve (data not shown) shows excellent *c*-axis orientation with narrow full width half maxima for all thin films. In terms of the Fe-doping trend on SRFO, it is clear from Fig. 1 that up to *x* = 0.20, the diffraction peaks of the thin films shift towards a lower 2*θ* position as the Fe content increases. This trend indicates that the lattice parameters and unit cell volume increase. In contrast, for *x* = 0.30, the peak of the thin film shifts towards the right relative to the position at *x* = 0.20, implying that the lattice constants decrease. This change in the lattice parameters of SRFO thin films induced by Fe doping can be explained in terms of possible substitution of Fe^3+^ ions rather than Fe^4+^ ions at Ru^4+^ sites, as discussed previously in detail [13]. From Fig. 1, it is also evident that for both *x* = 0.10 and 0.20 SRFO thin films, the diffraction peak shifts to a lower angle with decreasing deposition oxygen partial pressure, which implies an increase in the oxygen-vacancy content and an increase in the lattice parameters. An increase of the *c*-axis lattice parameter is a well-known characteristic of perovskite structures gaining oxygen vacancies [1].

**Fig. 2:**
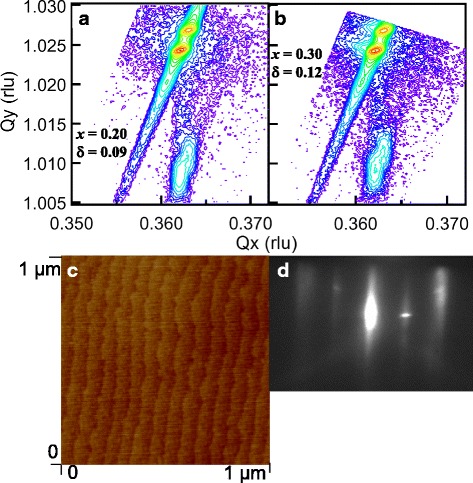
(Color online) X-ray RSM around the STO (103) planes for **a** SrRu_0.8_Fe_0.2_O_3.00 − 0.09_/STO (001) and **b** SrRu_0.7_Fe_0.3_O_3.00 − 0.12_/STO (001). **c** Surface morphology of SrRu_0.9_Fe_0.1_O_3.00 − 0.04_/STO thin film and **d** RHEED pattern of SrRu_0.9_Fe_0.1_O_3.00 − 0.04_/STO (001)

Reciprocal space mapping (RSM) is a powerful tool to investigate the epitaxial growth of thin films. Figure 2a, b shows the representative RSM of the (103) reflections of SrRu_0.8_Fe_0.2_O_3.00 − 0.09_ and SrRu_0.7_Fe_0.3_O_3.00 − 0.12_ thin films, respectively. (The RSM of other films was reported previously [13, 14].) The substrate and film peak can be easily identified in the RSM results. The diffraction peaks of both films are located right below the substrate peak, implying that the in-plane lattice parameters of both thin films are identical to that of the substrate. Based on the RSM images, it can be deduced that the thin films are compressively strained along the in-plane direction of the STO substrate.

The surface morphology of the SrRu_0.7_Fe_0.3_O_3.00 − 0.12_ thin film is shown in Fig. 2c. (The AFM images of other films were reported previously.) The SrRu_0.7_Fe_0.3_O_3.00 − 0.12_ film contains a step-terrace feature with root-mean-square roughness as low as 0.25 nm with atomic smoothness, consistent with the two-dimensional film growth revealed by our previous report [14]. The intensity of the spot was reported to oscillate in our previous report [14]. The image of the reflection of high-energy electron diffraction (RHEED) (Fig. 2d) during the film growth shows one bright spot, corresponding to an atomically flat film surface. In addition, the typical streak-like pattern observed throughout the deposition implies a layer-by-layer growth mode with high-quality crystallinity.

Table 1 summarizes the structural, transport, and magnetic properties of the epitaxial thin films used in the present study. Note that the oxygen-vacancy content listed in the table is estimated from the observed unit cell volume of the thin films, details of which were reported previously [13, 14]. From the unit cell volume of the SrRu_1 − *x*_Fe_*x*_O_3 − *δ*_ thin films, we can estimate the relative content of SrFeO_2.5_ and SrFeO_3.0_. When calculating the volume ratio (VR) and *δ* values, we neglect the sophisticated superstructure in SrFeO_*x*_ with 2.5 < *x* < 3.0 [23] and simply assume that our films consist of a mixture of three phases, i.e., SrRuO_3.0_, SrFeO_2.5_, and SrFeO_3.0_. Using linear estimation, a rough estimation of the VR (= unit cell volume of SrFeO_3_/(unit cell volume of SRFeO_2.5_ + SRFeO_3_) of SrFeO_3.0_ and SrFeO_2.5_ and oxygen-vacancy content (*δ* value) is performed. The stability of SrFeO_3.0_, i.e., the oxygen-rich phase, with respect to SrFeO_2.5_, i.e., the oxygen-poor phase, is the highest for the film with lower Fe doping of *x* = 0.10 and higher oxygen partial pressure during the growth process. The VR value is less than ~0.2 for SrRu_0.7_Fe_0.3_O_3 − 0.12_ and SrRu_0.8_Fe_0.2_O_3 − 0.09_, and the VR and *δ* values of SrRu_0.7_Fe_0.3_O_3 − 0.12_ are slightly larger than those of SrRu_0.8_Fe_0.2_O_3 − 0.09_. The XRD peak tends to shift to higher angles when the oxygen-vacancy content decreases and the VR increases. The effects of these two factors nearly cancel each other, resulting in almost the same unit cell volume for SrRu_0.7_Fe_0.3_O_3 − 0.12_ and SrRu_0.8_Fe_0.2_O_3 − 0.09_. The pseudo-cubic unit cell volumes of SrFeO_3_, SrFeO_2.5_, and STO substrates are 3.940, 3.850, and 3.905 Å, respectively. Thus, with increasing *x*, the SrFeO_3_ phase is promoted over the SrFeO_2.5_ phase, consequently reducing the VR.

**Table 1:** Structural, transport, and magnetic properties of SrRu_1 − *x*_Fe_*x*_O_3 − *δ*_ (*x* = 0.1, 0.2, and 0.3) thin films with different concentrations of oxygen vacancies

Film	*P* (O_2_) (mTorr)	Oxygen-vacancy content (*δ*)	VR	*ρ* (at 5 K) (μΩ cm)	*ρ* (300 K) (μΩ cm)	*T*_C_ (K)
SrRu_0.9_Fe_0.1_O_3 − *δ*_	100	0.04	0.35	360	616	115
	180	0.02	0.65	290	735	118
SrRu_0.8_Fe_0.2_O_3 − *δ*_	100	0.09	0.15	870	706	100
	180	0.07	0.37	570	735	100
SrRu_0.7_Fe_0.3_O_3 − *δ*_	100	0.12	0.18	13,805	1046	–

**Fig. 3:**
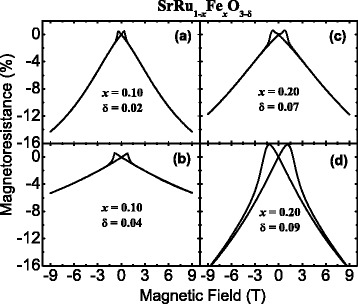
MR measurement configuration. MR versus applied magnetic field for all films at temperature *T* = 10 K. **a** SrRu_0.9_Fe_0.1_O_3.00 − 0.02_/STO (001) [13], **b** SrRu_0.9_Fe_0.1_O_3.00 − 0.04_/STO (001), **c** SrRu_0.8_Fe_0.2_O_3.00 − 0.07_/STO (001) [14], and **d** SrRu_0.8_Fe_0.2_O_3.00 − 0.09_/STO (001)

Figure 3 shows the magnetic-field dependences of the MR, defined as MR ≡ [*ρ*(*H*) − *ρ*(*H* = 0)]/*ρ*(*H* = 0) × 100%, for *x* = 0.10 and *x* = 0.20 thin films with low and high oxygen-vacancy content, where *ρ*(*H*) represents the resistivity at an applied magnetic field of *H*. Among the SrRu_1 − *x*_Fe_*x*_O_3 − *δ*_ thin films grown at a higher oxygen pressure (180 mTorr), the MR was higher for the thin film with *x* = 0.10 as compared with the thin film with *x* = 0.20 as shown in Fig. 3a, c, respectively [14]. As stated previously, we studied the intrinsic MR properties in Fe-doped SRO by fabricating epitaxial thin films and solving the grain boundary and A-site disorder problems of (Sr,La)(Ru,Fe)O_3_ polycrystals [12]. We also attempted to minimize the influence of oxygen vacancies on these thin films [13]. However, the obtained maximum MR value in SRFO thin films with low oxygen-vacancy content (max. MR of ~14.4% for *x* = 0.10) was considerably smaller than that observed in (Sr,La)(Ru,Fe)O_3_ polycrystals (max. MR of ~45% for *x* = 0.30) [12]. A notable finding for the MR of (Sr,La)(Ru,Fe)O_3_ polycrystals was that the MR is highest for the sample in which the zero-field resistivity shows the highest value in the doping range of 0.10 ≤ *x* ≤ 0.30. Thus, we attempted to confirm this trend in our epitaxial thin films by measuring the MR of SrRu_1 − *x*_Fe_*x*_O_3 − *δ*_ (*x* = 0.10 and 0.20) thin films having more oxygen vacancies, as shown in Fig. 3b, d. In these samples, the MR increases to 16% as the Fe-doping concentration increases from 10 to 20%. Nonetheless, this value is still smaller than that observed in (Sr, La) (Ru,Fe) O_3_ polycrystals (MR of ~45%).

Regarding the MR trend of *x* = 0.10 thin films, the MR value of the thin film with a higher number of oxygen vacancies (SrRu_0.9_Fe_0.1_O_3 − 0.04_) is smaller than the MR of the thin film with a lower oxygen-vacancy content (SrRu_0.9_Fe_0.1_O_3 − 0.02_). Both films show metallic behavior in terms of resistivity, *ρ*(*T*), down to 10 K as reported previously, the temperature at which MR measurements were conducted. In contrast, the MR of SrRu_0.8_Fe_0.2_O_3 − 0.09_ is the highest among the four films, as shown in Fig. 3. This film features greater Fe doping and a higher oxygen-vacancy content.

**Fig. 4:**
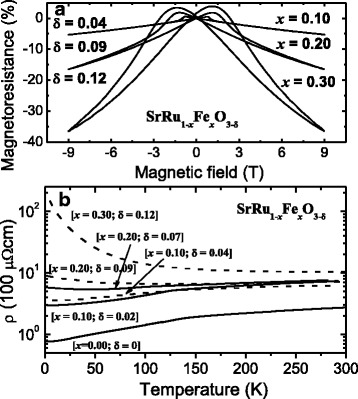
**a** MR versus applied magnetic field for SrRu_1-*x*_Fe_*x*_O_3.00 − *δ*_/STO thin films at temperature *T* = 10 K. **b** Temperature-dependent resistivity, *ρ*(*T*), for SrRu_1 − *x*_Fe_*x*_O_3 − *δ*_/STO (001). The *dashed* and *straight lines* represent results from our previous reports [13, 14]

Thus, the value of MR at 10 K seems to increase with an increase in Fe doping and oxygen-vacancy content for semiconducting thin films, while it seems to decrease with increasing oxygen-vacancy content for metallic thin films. In this context, a larger MR is expected for a thin film having more oxygen vacancies and showing more semiconducting behavior. To test this hypothesis, we prepared a thin film of SrRu_0.7_Fe_0.3_O_3 − 0.12_ and compared the MR for a series of SrRu_1 − *x*_Fe_*x*_O_3 − *δ*_ films grown at a lower oxygen partial pressure, as presented in Fig. 4a. As predicted, the MR value for the SrRu_0.7_Fe_0.3_O_3 − 0.12_ thin film increases by more than two times relative to the MR value of the SrRu_0.8_Fe_0.2_O_3 − 0.09_ thin film.

The origin of the high MR in our SRFO thin films, especially at lower temperature, is not fully known. The MR, however, is not related to that observed near the paramagnetic–ferromagnetic–semiconductor–metal transition in manganite materials [24], where the MR is sharply peaked around ferromagnetic transition temperature, *T*_*C*_. Herranz et al. explained the difference by the absence of Jahn-Teller effect in Ru^4+^ ion, whereas Jahn-Teller effect is present in Mn^3+^ ion [25]. SRO is an itinerant ferromagnet, whose magnetic properties are essentially tied to the electronic band coming from the hybridization of the Ru 4*d* orbitals and the O 2*p* orbitals. Since Ru^4+^ (*t*_2*g*_^4^ *e*_*g*_^0^) is not a Jahn-Teller ion, it is not expected to have an enhancement of the MR near *T*_C_. High MR is observed in our SRFO thin films at a temperature far below *T*_*C*_. Similar results have been obtained in other reports on SRO thin films and SRO bulk materials [25–27]. For example, Herranz et al. studied the temperature dependence of MR of SRO thin films and they observed highest MR at low temperature around 10 K, and then, the MR steadily decreased with increase in temperature [25]. They did not observe any enhancement of MR around *T*_*C*_. They attributed the observation of high MR at low temperature to spin-orbit coupling [25]. In another study, the observation of high MR at low temperature in SRO thin film was attributed to an orbital contribution [26]. The observation of high MR at low temperature in bulk Mn-doped SRO was related to field-induced magnetization [27]. From these discussions, it seems that the observation of high MR in our SRFO thin films may be due to orbital contributions or due to spin-orbit coupling. Further work is clearly required to establish the exact mechanism of MR in SRFO thin films.

The temperature dependence of the zero-field resistivity of the SrRu_1 − *x*_Fe_*x*_O_3 − *δ*_ (*x* = 0.1, 0.2, 0.3) thin films deposited at high and low oxygen partial pressures is shown in Fig. 4b. It might initially seem that the dramatic change in the resistivity of the SrRu_1 − *x*_Fe_*x*_O_3 − *δ*_ thin films is due to Fe doping. However, if we consider the resistivity of the SRFO thin films with the same *x* deposited at higher and lower oxygen partial pressures, we can clearly observe that resistivity changes appreciably due to the increase of oxygen vacancies. Now, let us compare the efficiency of resistivity change by the change in oxygen content and replacement of Ru ion by Fe ion from SrRuO_3_ to SrRu_0.7_Fe_0.3_O_3 − 0.12_. The oxygen content changes from 3.0 to 2.88 (4% change), while the replacement of Ru ion by Fe ion was 30%. Thus, a 4% change in oxygen contents seems to bring about similar variation to MR as 30% replacement of Ru ion by Fe ion. Thus, oxygen-vacancy content has significantly more impact on MR than replacement of Ru ion by Fe ion on MR. The higher resistivity of the SrRu_0.80_Fe_0.20_O_3 − 0.09_ thin films than of the SrRu_0.90_Fe_0.10_O_3 − 0.02_ thin films can be attributed primarily to the presence of a higher number of oxygen vacancies in the *x* = 0.20 thin film. From Fig. 4b, it is evident that the metal–insulator transition temperature shifts to a lower temperature range with an increase in both Fe doping and oxygen-vacancy content, implying that both factors have a major influence on the *T*_C_ of the material. With an increase in Fe doping, the *ρ*(*T*) behavior in SRFO thin films changes from the metallic (*x* = 0.10) regime to the semiconducting (*x* = 0.30) regime. Furthermore, in the intermediate Fe-doping range (*x* = 0.20), the thin film exhibits metallic behavior at high temperatures and insulating behavior at low temperatures. Out of all the SRFO thin films, the SrRu_0.7_Fe_0.3_O_3 − 0.12_ thin film shows the most prominent semiconducting behavior and has the highest oxygen-vacancy content, as predicted previously, and thus displays the highest MR value.

## Conclusions

In this study, we fabricated high-quality single-phase SrRu_1 − *x*_Fe_*x*_O_3 − *δ*_ epitaxial thin films with different oxygen vacancies by PLD. We observed a metal–insulator transition for films with *x* = 0.20 and *x* = 0.10. In contrast, the film with *x* = 0.30 displayed an insulating behavior over the entire temperature range of 2–300 K. Here, the effect of oxygen-vacancy content played a major role in determining the magneto-transport properties of the SRFO epitaxial thin film. With the increase in Fe-doping concentration, the oxygen-vacancy content increased, which in turn increased the negative MR. For the low-doping case of *x* = 0.10, the MR was higher for the film with fewer oxygen vacancies. The SrRu_0.7_Fe_0.3_O_3 − 0.12_ film with the largest MR (~36.4%) exhibited the largest oxygen-vacancy content (*δ* = 0.12) and the most prominent semiconducting behavior.

## Abbreviations

AFM: Atomic force microscopy
HRXRD: High-resolution X-ray diffraction
MR: Magnetoresistance
PLD: Pulsed laser deposition
RHEED: Reflection of high-energy electron diffraction
RSM: Reciprocal space mapping
SLRFO: (Sr_1 − *x*_La_*x*_)(Ru_1 − *x*_Fe_*x*_)O_3_
SRFO: SrRu_1 − *x*_Fe_*x*_O_3_
SRO: SrRuO_3_
STO: SrTiO_3_
*T*_*C*_: Curie temperature
VR: Volume ratio
